# 4-Pyridylnitrene and 2-pyrazinylcarbene

**DOI:** 10.3762/bjoc.9.85

**Published:** 2013-04-17

**Authors:** Curt Wentrup, Ales Reisinger, David Kvaskoff

**Affiliations:** 1 School of Chemistry and Molecular Biosciences, The University of Queensland, Brisbane, Qld 4072, Australia

**Keywords:** carbene–nitrene interconversion, diazepines, flash vacuum thermolysis, matrix photochemistry, nitrile ylides, reactive intermediates

## Abstract

Both flash vacuum thermolysis (FVT) and matrix photolysis generate 2-diazomethylpyrazine (**22**) from 1,2,3-triazolo[1,5-*a*]pyrazine (**24**). FVT of 4-azidopyridine (**18**) as well as of **24** or 2-(5-tetrazolyl)pyrazine (**23**) affords the products expected from the nitrene, i.e., 4,4’-azopyridine and 2- and 3-cyanopyrroles. Matrix photolyses of both **18** and **24** result in ring expansion of 4-pyridylnitrene/2-pyrazinylcarbene to 1,5-diazacyclohepta-1,2,4,6-tetraene (**20**). Further photolysis causes ring opening to the ketenimine **27**.

## Introduction

**Scheme 1:**
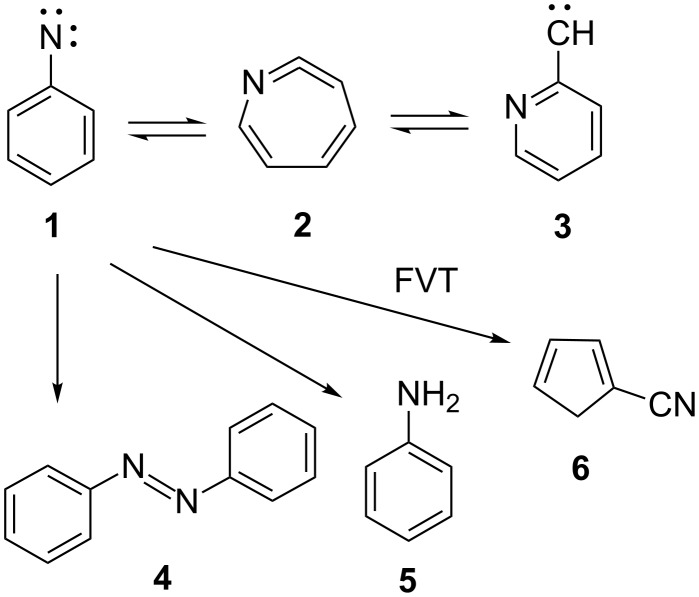
Phenylnitrene–2-pyridylcarbene rearrangement.

The carbene–nitrene interconversion exemplified with phenylnitrene (**1**) and 2-pyridylcarbene (**3**) has been described in considerable detail [1–3]. The three species **1**–**3** have all been observed directly by IR or ESR spectroscopy in low-temperature matrices. The normal reaction products of phenylnitrene are azobenzene (**4**) and aniline (**5**), but under forcing flash vacuum thermolysis (FVT) conditions cyanocyclopentadiene **6** is formed (Scheme 1). Several other carbene–nitrene rearrangements have been reported [3–5].

**Scheme 2:**
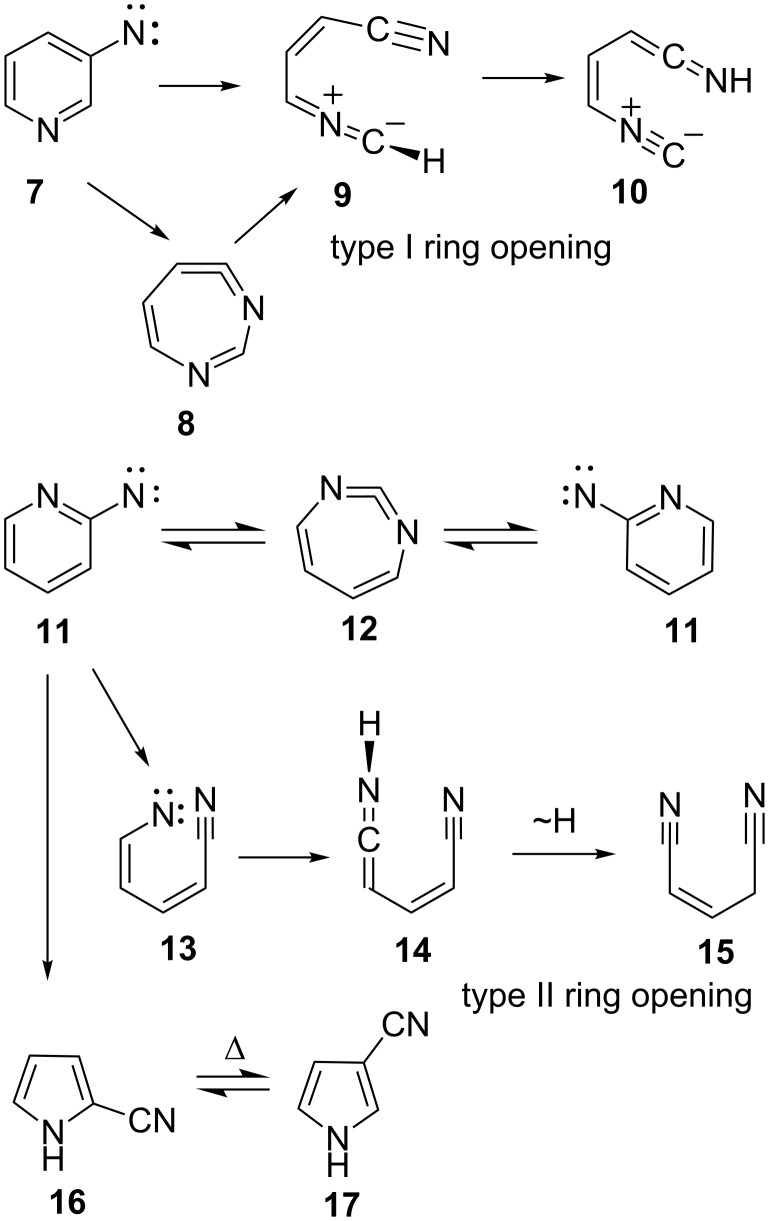
Type I and type II ring opening and ring expansion in 3- and 2-pyridylnitrenes, respectively.

In addition to the ring expansion (**1**–**2**–**3**), two ring opening reactions have been investigated in recent years. Type I ring opening takes place in nitrenes possessing a 1,3-relationship between a nitrene centre and a ring nitrogen atom and leads to nitrile ylides, such as **9** in the case of 3-pyridylnitrene (**7**), whereby the actual ring opening may take place in either the nitrene itself or the ring-expanded ketenimine **8** (Scheme 2) [6–8]. A 1,7-H shift finally converts the nitrile ylide **9** to the open-chain ketenimine **10** (Scheme 2). 3-Pyridylcarbene undergoes analogous Type I ylidic ring opening to an ethynylvinylnitrile imine [6]. Type II ring opening is diradicaloid and proceeds via an open-chain vinylnitrene or biradical **13** in nitrenes such as 2-pyridylnitrene (**11**). This leads to minor amounts of ketenimines **14** or glutacononitriles **15** beside the main product, the cyclic carbodiimide **12**, which is formed in a degenerate rearrangement (**11**≡**11**). The major end products of FVT are the cyanopyrroles **16** and **17** (Scheme 2) [3,9–11].

## Results and Discussion

**Scheme 3:**
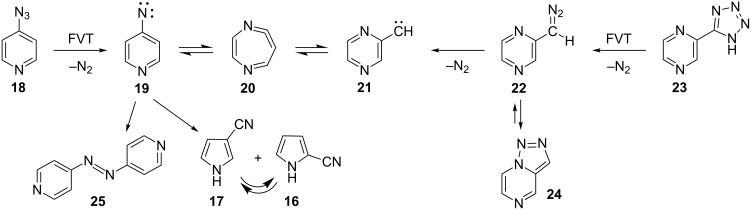
FVT reactions of 4-azidopyridine (**18**), 2-(5-tetrazolyl)pyrazine (**23**) and triazolo[1,5-*a*]pyrazine (**24**).

Here, we report details of the FVT as well as matrix photolysis reactions of 4-azidopyridine (**18**) and the isomeric triazolo[1,5-*a*]pyrazine (**24**) and its precursor, 2-(5-tetrazolyl)pyrazine (**23**). Furthermore, we report evidence for ring expansion as well as ring opening of 4-pyridylnitrene (**19**) and 2-pyrazinylcarbene (**21**) (Scheme 3). Mild FVT of **18** at 400 °C, even in high vacuum, affords 4,4’-azopyridine (**25**) in 54% yield [12]. This kind of reaction is typically ascribed to the dimerization of triplet nitrenes. Most importantly, FVT of the pyrazinylcarbene precursor **23** also affords a small amount of 4,4’-azopyridine (**25**), thus implying a rearrangement of carbene **21** to nitrene **19** (Scheme 3). Presumably, this takes place via the diazacycloheptatetraene, **20**, which was not directly observed under the FVT conditions. It was, however, readily observed under photolysis conditions, as described below. Since nitrenes are intrinsically more stable than the isomeric carbenes [3,12–13], a rearrangement of **21** to **19** is perfectly reasonable.

In the case of phenylazide, mild FVT results in the formation of azobenzene (**4**) (Scheme 1), but violent FVT, where the pressure is allowed to rise to ca. 1 mbar due to a shock wave induced by rapid distillation of the azide into the furnace under high vacuum, results in ring contraction to cyanocyclopentadiene **6** [12]. Similar pyrolysis of 4-azidopyridine results in ring contraction to a mixture of 2- and 3-cyanopyrroles **16** and **17**. Because the ring contraction is highly exothermic, the product will be chemically activated [14], and therefore interconversion of the two cyanopyrroles cannot be avoided. Only a minute trace of 4,4’-azopyridine is formed under these conditions. The total yield of the cyanopyrroles is low because of heavy charring inside the pyrolysis tube. The exact mechanisms of ring contraction in arylnitrenes are under investigation, but in the case of 2-pyridylnitrene (Scheme 2) three routes to the cyanopyrroles have been described [10], as has their thermal interconversion [9–10]. The analogous, concerted ring contraction of phenylnitrene to 5-cyanocyclopentadiene has a calculated barrier of ca. 30 kcal/mol [15].

**Figure 1:**
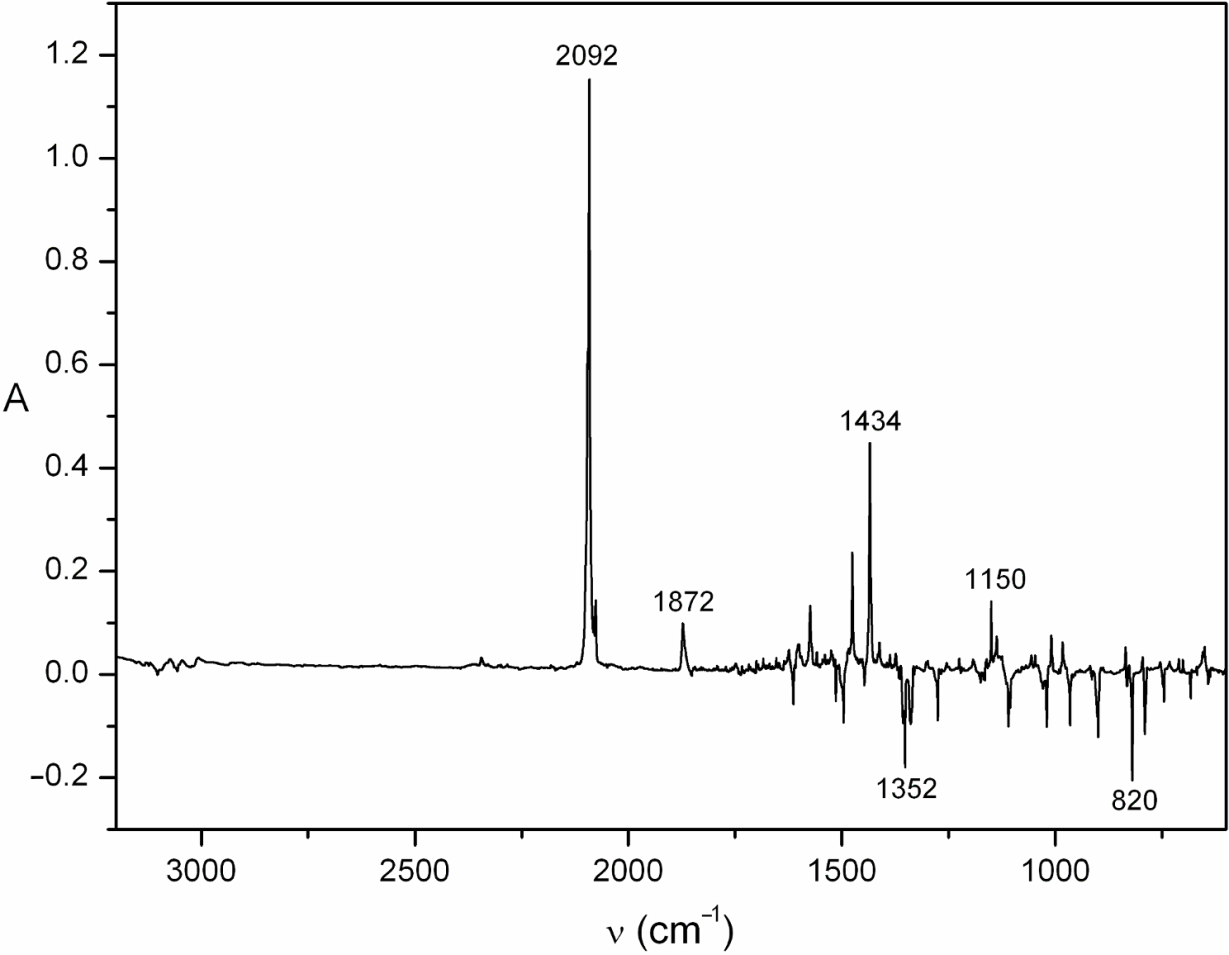
Difference-IR spectrum of 2-diazomethylpyrazine (**22**) (positive peaks) in Ar matrix at 7 K, obtained by photolysis of triazolopyrazine **24** at 290 nm for 10 min (negative peaks). Spectrum assigned to **22**: 795, 835, 918, 1009, 1056, 1150, 1299, 1434, 1476, 1574, 2076 and 2092 cm^−1^; the latter two absorptions are assigned to the two s-*E* and s-*Z* conformations of the diazo group. The peak at 1872 cm^−1^ is due to the formation of a small amount of the photoproduct **20**. See the Supporting Information File 1, Figure S1 for a full spectrum of **24** with peak listing.

FVT of the tetrazole **23** at 400 °C/10^−4^ mbar causes loss of N_2_ and formation of 2-diazomethylpyrazine (**22**), which is easily observed directly by its strong absorption at 2080 cm^−1^ when the pyrolysate is isolated neat in liquid N_2_ at −196 °C, or at 2092 cm^−1^ upon isolation in an Ar matrix at 10 K. The diazo compound is also formed on matrix photolysis of the triazole (Figure 1). Compound **22** can exist as s-*Z* and s-*E* conformers. One conformer dominates, absorbing strongly at 2092 cm^−1^; the other, minor absorption is at 2076 cm^−1^ (Figure 1). Warming the neat diazo compound to ca. −30 °C causes ring closure to triazole **24**. Compound **24** can be isolated in up to 20% yield in preparative pyrolysis of **23** [16]. Further FVT of either **23** or **24** at ≥450 °C/10^−3^ mbar results in the formation of approximately equal amounts of the cyanopyrroles **16** and **17** and azo compound **25**, but the overall yield is again poor, ca. 30%, due to heavy charring in the pyrolysis tube. It is worth noting that ethynylimidazoles, which could be thought of as ring-contraction products of carbene **21**, were not detectable.

**Figure 2:**
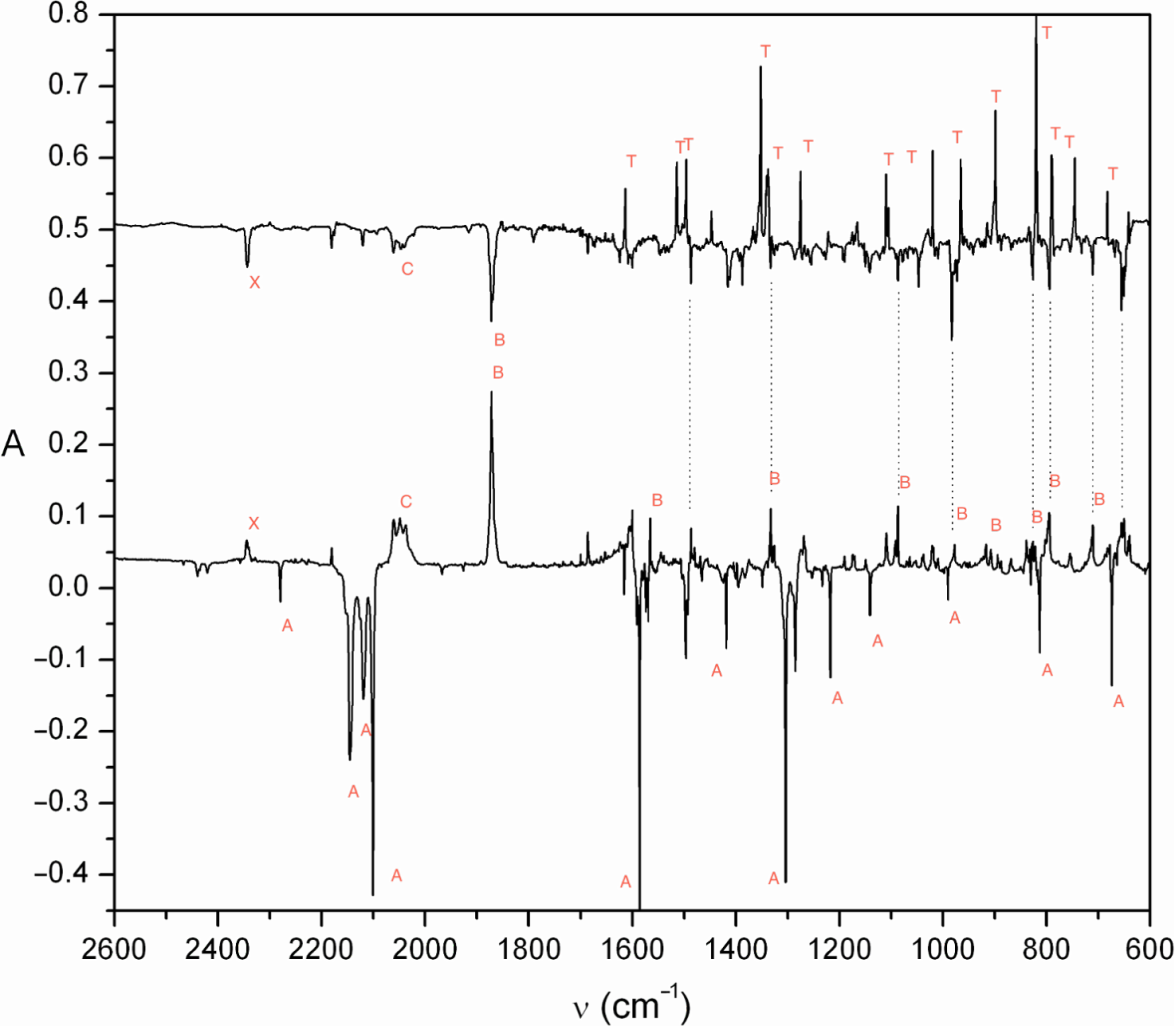
Ar matrix IR-difference spectra showing the products of broadband UV photolysis of 4-azidopyridine (**18**) (bottom, bands labelled A) and 1,2,3-triazolo[1,5-*a*]pyrazine (**24**) (top, bands labelled T). In the photolysis of **18** the negative peaks (A) are due to the subtracted spectrum of azide **18**; the positive peaks are due to the products **20** (B) and **27** (C). In the photolysis of **24** the negative peaks are the products **20** and **27**; the positive peaks are due to the subtracted spectrum of the triazole **24** (T). A small peak labelled X at 2340 cm^−1^ is due to CO_2_. Ordinate in arbitrary absorbance units. See Figure S1–S3 in Supporting Information File 1 for full spectra of **18**, **20** and **24** with peak listings.

Photolysis of either 4-azidopyridine (**18**) or triazole **24** in an Ar matrix at 7–10 K causes efficient ring expansion to the cyclic seven-membered ring ketenimine **20** as observed by IR spectroscopy (Figure 2). In the case of triazole **24**, efficient ring opening to the diazo compound **22** happens first (Figure 1), so the latter is the immediate precursor of **20**. The IR spectrum of the triazole **24** is shown in Figure S1, Supporting Information File 1.

**Figure 3:**
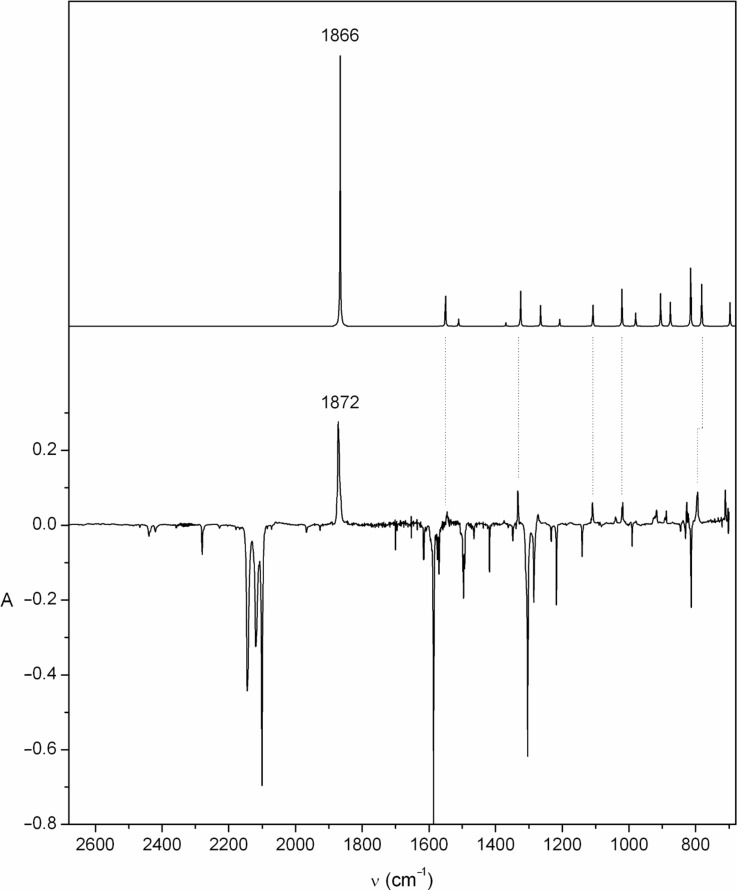
Top: calculated IR spectrum of **20** at the B3LYP/6-31G* level (wavenumbers scaled by 0.9613): ν’ (relative intensity) 697 (9), 782 (16), 815 (22), 876 (9), 905 (12), 980 (5), 1021 (14), 1108 (8), 1208 (3), 1265 (8), 1325 (13), 1511 (3), 1550 (11), 1866 (100) cm^−1^. Ordinate in arbitrary absorbance units. Bottom: Ar matrix IR-difference spectrum of diazacycloheptatetraene **20** (ν’ 710, 794, 826, 887, 1028, 1109, 1272, 1332, 1545, 1872 cm^−1^), formed by photolysis of 4-azidopyridine (**18**) (3 min broadband UV; positive bands due to **20**; negative bands due to **18**). See Figures S2 and S3 in Supporting Information File 1 for full spectra of **18** and **20** with peak listings.

It is clearly seen in Figure 2 that the two starting materials **18** and **24** afford nearly identical product mixtures, and the absorptions of ketenimine **20** account for the majority of the spectrum, including the very strong cumulenic absorption at 1872 cm^−1^. This cumulene **20** is obtained nearly pure after short photolysis times (Figure 3).

**Figure 4:**
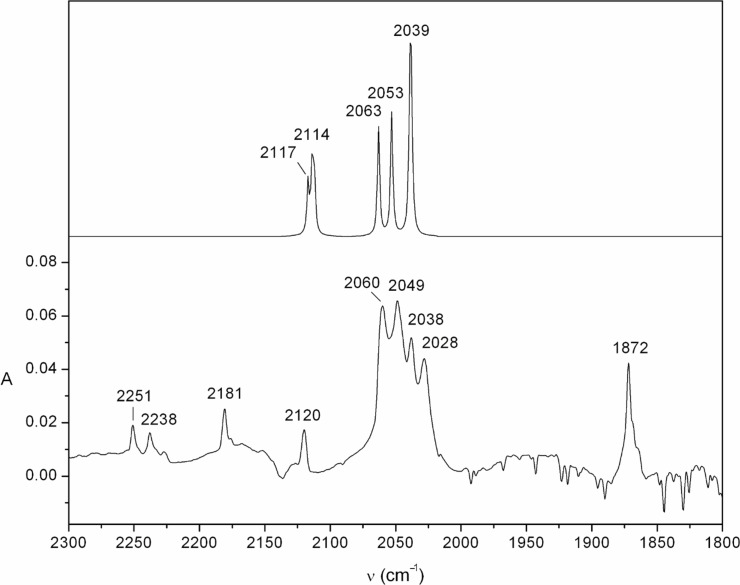
Bottom: IR spectrum from the matrix photolysis of azide **18** after the azide has been depleted completely (10 min broadband UV, followed by 30 min low pressure Hg lamp at 254 nm). Top: the calculated absorptions of the four isomers of the open chain ketenimine **27** (1:1:1:1 ratio of the s-*E*,*E*; s-*E*,*Z*; s-*Z*,*E*; and s-*Z*,*Z* isomers). The calculated peak at 2039 cm^−1^ is due to the overlapping peaks of two isomers at 2039 and 2040 cm^−1^. See Figure S6 and Figure S7 in Supporting Information File 1 for structures and spectra of the individual isomers. Calculated spectra are at the B3LYP/6-31G* level (wavenumbers scaled by 0.9613). The 254 nm photolysis causes a faster rearrangement of **20** to **27**; broadband UV photolysis achieves a similar change in ca. 60 min.

**Scheme 4:**
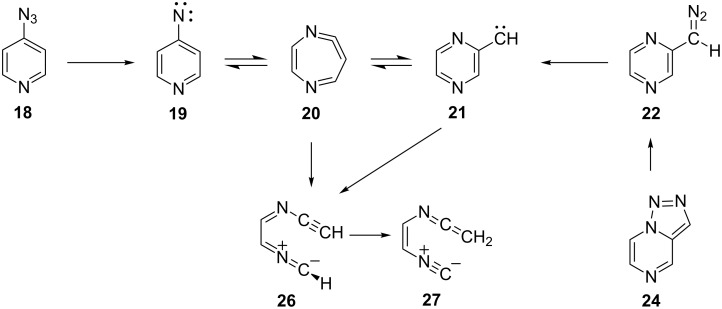
Photolysis reactions of azide **18** and triazole **24** in Ar matrix.

Further photolysis, especially at higher energy (254 nm), causes formation of a second product with prominent signals in the IR spectrum at 2028, 2038, 2049, 2060 and 2120 cm^−1^ (Figure 4 and Supporting Information File 1, Figure S4 and Figure S5). The group of bands at 2028–2060 cm^−1^ is ascribed to the ketenimine moiety (N=C=C), and the band at 2120 cm^−1^ to the isocyanide (NC) function in the ring-opened *N-*(isocyanovinyl)ketenimine (**27**) (Scheme 4). The latter band is not seen in the the pyridyl azide photolysis in Figure 2 because of masking by the strong, negative azide bands. However, the 2120 cm^−1^ band is clearly seen later in the photolysis when most of the azide has been consumed (see Figure 4 and Supporting Information File 1, Figure S5). The temporal evolution of the azide photolysis reveals that the cyclic ketenimine **20** is formed first, followed by the open chain ketenimine **27** (Figure S3, Supporting Information File 1). It should be noted that ketenimine **27** can exist as four different s-*E* and s-*Z* conformers, with slightly different IR absorptions (the calculated spectra are shown in Figure 4 and Figure S6 and Figure S7, Supporting Information File 1). The experimental spectra indicate that all four conformers of **27** are formed, giving rise to bands at 2028–2060 cm^−1^, i.e., there is enough energy available to effect *E–*Z and s-*E*–s-*Z* isomerization in the matrix. Also the isocyanide band at 2120 cm^−1^ shows splitting, but this is less well resolved (Figure 4). All this makes it difficult to assign other, much weaker, peaks in the IR spectra to compound **27**. However, the following bands, which are not ascribed to the cyclic ketenimine **20**, can be assigned to **27**: Observed wavenumbers and, in parentheses, the calculated harmonic frequencies of the conformers of **27** at the B3LYP/6-31G* level, scaled by 0.9613: 711 (691–703), 1300 (1256–1290), 1380 (1373–1377), 1608–1624 (1612–1634), 2028, 2038, 2049, 2061 (2038–2063), 2119–2124 (2112–2117) cm^−1^ (see Supporting Information File 1 for computational details).

We postulate that the rearrangement to **27** occurs via the unobserved nitrile ylide intermediate **26** (Scheme 4). The calculated IR spectra of the four s-*E* and s-*Z* conformers of **26** are shown in Figure S8 and Figure S9 in Supporting Information File 1. Related nitrile ylides are known to undergo very facile 1,7-H shifts (see, e.g., Scheme 2) [3,6]. Both the nitrile ylide and the ring-expanded ketenimine were observed in the corresponding benzannelated systems, viz 4-azidoquinoline and 2-diazomethylquinoxaline [17], and the ylidic ring opening is very facile in 3-pyridylcarbene [6]. Thus, there is good precedence for transformations of the type **26**→**27** (Scheme 4).

## Conclusion

2-Pyrazinylcarbene (**21**) rearranges to 4-pyridylnitrene (**19**) on FVT as evidenced by the isolation of 4,4’-azopyridine (**25**). Both **19** and **21** undergo ring expansion to the diazacycloheptatetraene **20** on UV photolysis. Compound **20** is clearly identified by its matrix IR spectrum, particularly the strong cumulenic stretch at 1872 cm^−1^. Further photolysis causes the gradual depletion of **20** and formation of the ring opened ketenimine **27**. The latter reaction indicates ring opening of either cumulene **20** or carbene **21** to the nitrile ylide **26** followed by a rapid 1,7-H shift to **27**.

In summary, 4-pyridylnitrene **19** behaves much like phenylnitrene **1**. The thermal chemistry of both the nitrene **19** and the carbene **21** is dominated by nitrene reactions (giving azopyridine and cyanopyrroles), because the carbene rearranges to the lower energy nitrene. However, the 2-pyrazinylcarbene **21** possesses a 1,3-relationship between the carbene centre and a ring nitrogen atom like in 3-pyridylcarbene [6] and therefore undergoes ylidic ring opening. Thanks to the reversible carbene–nitrene rearrangement (**19** 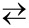 **20** 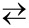 **21**) formation of the ylide **26** and hence the ketenimine **27** becomes the dominant reaction under the conditions of matrix photolysis.

## Experimental

### General

The apparatus and procedures for preparative FVT [18] and for Ar matrix isolation [11,19–20] were as previously described. KBr and CsI windows were used for IR spectroscopy. FVT products were isolated in liquid nitrogen (77 K) in the preparative thermolyses, and at 22–25 K in Ar matrices for IR experiments. IR spectra of the Ar matrices were measured at 7–10 K with a resolution of 1 cm^−1^. Photolyses were done through quartz by using a 75 W low-pressure Hg lamp (254 nm) or a 1000 W high pressure Hg/Xe lamp equipped with a monochromator and appropriate filters. A water filter was used to remove infrared radiation.

#### Materials

4-Azidopyridine (**18**) was prepared from 4-bromopyridine and NaN_3_ in 10% ethanol under reflux for 8 h by adaptation of a literature method [21]. 1,2,3-Triazolo[1,5-*a*]pyrazine (**24**) was prepared according to a literature procedure [16].

#### FVT of 4-azidopyridine (**18**)

The azide (0.50 g) was distilled into the FVT apparatus from a sample flask held at 35 °C and thermolysed at 400 °C/10^−2^–10^−3^ mbar in the course of 1 h. 4,4’-Azopyridine (**25**) (54% yield; red solid, mp 107–108 °C) was identified by comparison with a sample prepared according to den Hertog et al. (mp 107.5 °C) [22]. Traces of unreacted azide **18** and 2- and 3-cyanopyrroles **16** and **17**were detected by GC–MS and IR spectroscopy.

“Violent pyrolysis” was achieved by holding the sample flask at 100 °C. The rapid distillation causes a shock wave and a pressure increase to 1 mbar under continuous pumping with a two-stage oil pump capable of a base vacuum of 10^−4^ mbar. The whole reaction is finished in a matter of seconds. The 2- and 3-cyanopyrroles **16** and **17** were isolated in a ratio of ca. 1:1 in a total yield of 10% as analysed by GC. Only a trace of 4,4’-azopyridine (**25**) was formed under these conditions. There was heavy charring inside the pyrolysis tube.

#### Photolysis of 4-azidopyridine (**18**)

The azide (5–10 mg) was evaporated from a reservoir at −30 to −40 °C and codeposited with Ar at 25 K. After cooling to 7 K, the spectrum was recorded (Figure S2, Supporting Information File 1): IR (Ar, 7 K) 813, 1271, 1303, 1586, 2100, 2119, 2145, 2280 cm^−1^.

The azide matrix was irradiated with broadband UV light from the high-pressure Xe/Hg lamp, at 290 nm using the monochromator, or at 254 nm using the low-pressure Hg lamp. The resulting product-difference IR spectra are shown in Figure 2, Figure 3 and Figure 4. The temporal evolution of the spectrum under continuous broadband photolysis at 7 K is shown in Figure S3, Figure S4 and Figure S5, Supporting Information File 1.

#### FVT of 2-(5-tetrazolyl)pyrazine (**23**)

The preparation of triazolopyrazine **24** by FVT of **23** at 400 °C has been described previously [19]. FVT of **23** at 450 °C/10^−3^ mbar caused formation of triazole **24**, 4,4’-azopyridine (**25**) and the 2- and 3-cyanopyrroles **16** and **17** in a ca. 2:1:2:2 ratio and a total yield of ca. 30%. There was heavy charring inside the pyrolysis tube. The cyanopyrroles were isolated by distillation and separated by GC [9]. Compounds **24** and **25** were separated by chromatography on alumina, eluting with CHCl_3_. The products were identified by comparison of IR, NMR and mass spectra with those of authentic materials [9,22]. Careful searches for ethynylimidazoles were negative.

#### Photolysis of 1,2,3-triazolo[1,5-*a*]pyrazine (**24**)

The triazole was purified by recrystallization from hexane/ethyl acetate and then sublimed from a sample tube held at 50–60 °C and codeposited with Ar at 25 K. After cooling to 7 K, the spectrum was recorded (Supporting Information File 1, Figure S1): IR (Ar, 7 K) 642, 683, 745, 790, 820, 899, 966, 1020, 1105, 1110, 1165, 1275, 1338, 1352, 1447, 1496, 1514, 1613, 3057 cm^−1^. Irradiation at 290 nm for 10 min afforded 2-diazomethylpyrazine with principal absorptions at 2092 cm^−1^ (major conformer) and 2076 cm^−1^ (minor conformer) (Figure 1): IR (Ar, 7 K) 795, 835, 918, 1009, 1056, 1150, 1299, 1434, 1476, 1574, 2076, 2092 cm^−1^. A small amount of the photoproduct **20** also appeared (1872 cm^−1^; Figure 1). Further photolysis afforded the product spectrum shown in Figure 2 and in Supporting Information File 1, Figure S3, Figure S4 and Figure S5.

## Supporting Information

File 1 Additional matrix IR spectra of **18**, **24**, and their photolysis products, calculated IR spectra of **20**, **26** and **27**, and computational details.
