# Supplementary material for: 4-Pyridylnitrene and 2-pyrazinylcarbene
Source: Beilstein J Org Chem. 2013 Apr 17;9:754–60. doi: 10.3762/bjoc.9.85 (PMC3678510; doi:10.3762/bjoc.9.85)
Supplement: File 1 — Additional matrix IR spectra of 18, 24, and their photolysis products, calculated IR spectra of 20, 26 and 27, and computational details. [file Beilstein_J_Org_Chem-09-754-s001.pdf]

Supporting Information

for

4-Pyridylnitrene and 2-pyrazinylcarbene

Curt Wentrup*, Ales Reisinger and David Kvaskoff

Address: School of Chemistry and Molecular Biosciences, The University of Queensland, Brisbane, Qld 4072, Australia

Email: Curt Wentrup* - wentrup@uq.edu.au

* Corresponding author

Additional matrix IR spectra of **18**, **24**, and their photolysis products, calculated IR spectra of **20**, **26** and **27**, and computational details

Contents:

Figure S1 Ar matrix IR spectrum of 1,2,3-triazolo[1,5-*a*]pyrazine **24**	S2
Figure S2 Ar matrix IR spectrum of 4-azidopyridine **18**	S3
Figure S3 IR-difference spectrum showing **20** and azide **18**	S3
Figure S4 Temporal evolution of photolysis of 4-azidopyridine **18**	S4
Figure S5 IR-difference spectrum resulting from the 254 nm-photolysis of **20** to **27**.	S5
Figure S6 Calculated IR spectra of two *E*-conformers of *N*-(isocyanovinyl)ketenimine **27**	S6
Figure S7 Calculated IR spectra of two *Z*-conformers of *N*-(isocyanovinyl)ketenimine **27**	S7
Figure S8 Calculated IR spectra of two s-*E*-conformers of ethinylimino-nitrile ylide **26**	S8
Figure S9 Calculated IR spectra of two s-*Z*-conformers of ethinylimino-nitrile ylide **26**	S9
Computational method, Cartesian coordinates, energies and harmonic vibrations	S9
References	S20

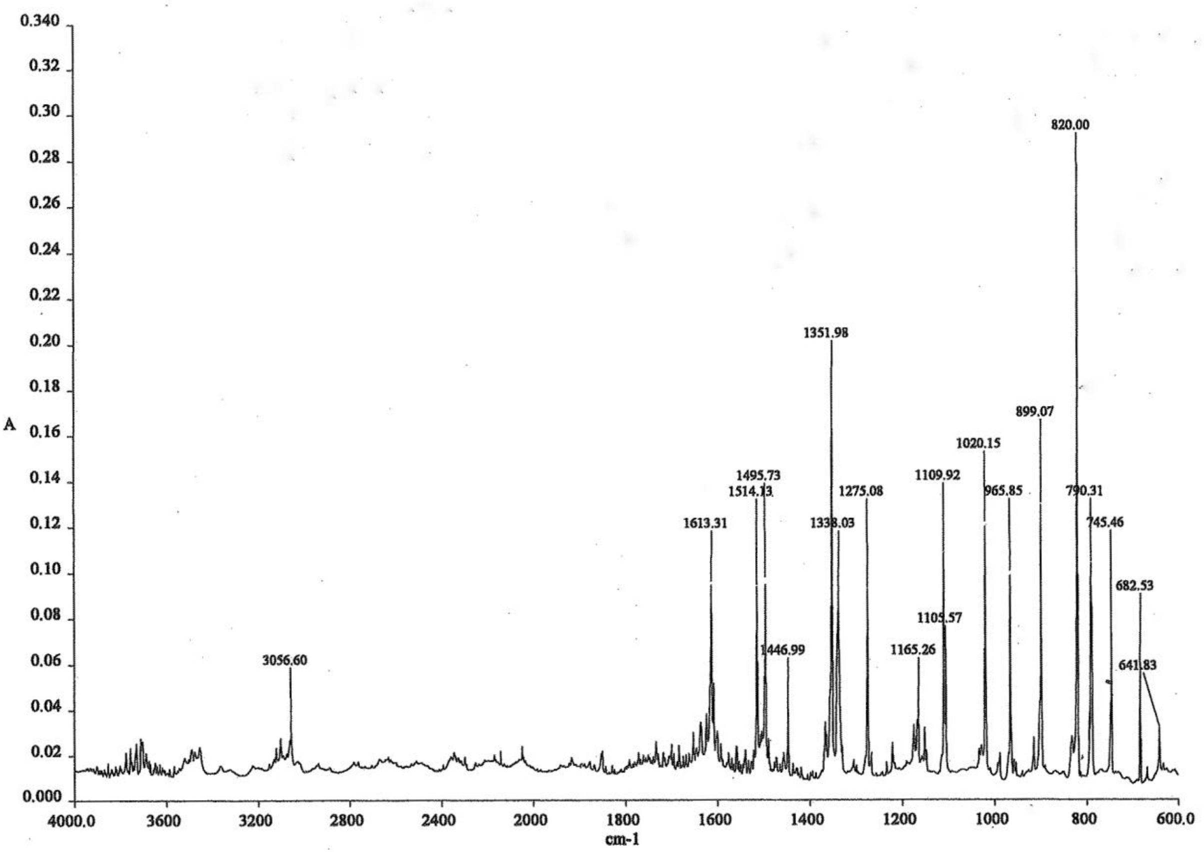

Figure S1: IR spectrum of 1,2,3-triazolo[1,5-*a*]pyrazine (**24**) matrix isolated in Ar at 7 K.

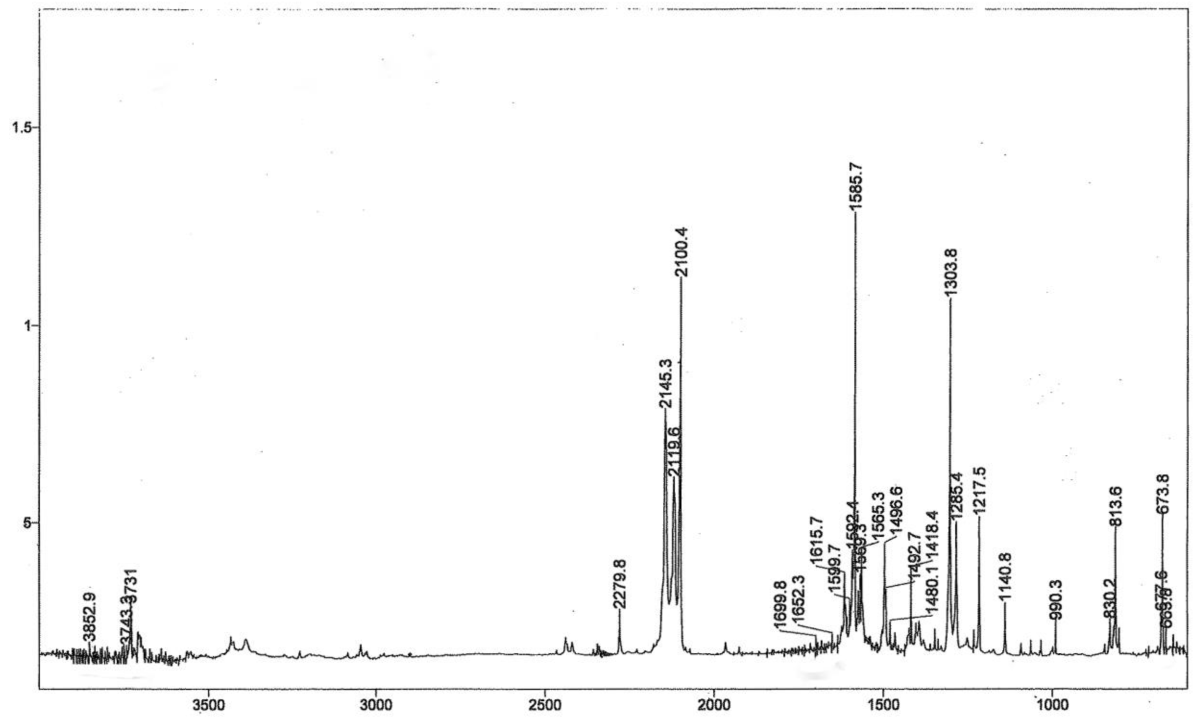

Figure S2: IR spectrum of 4-azidopyridine (**18**) matrix isolated in Ar at 7 K. Abscissa in cm^{-1}.

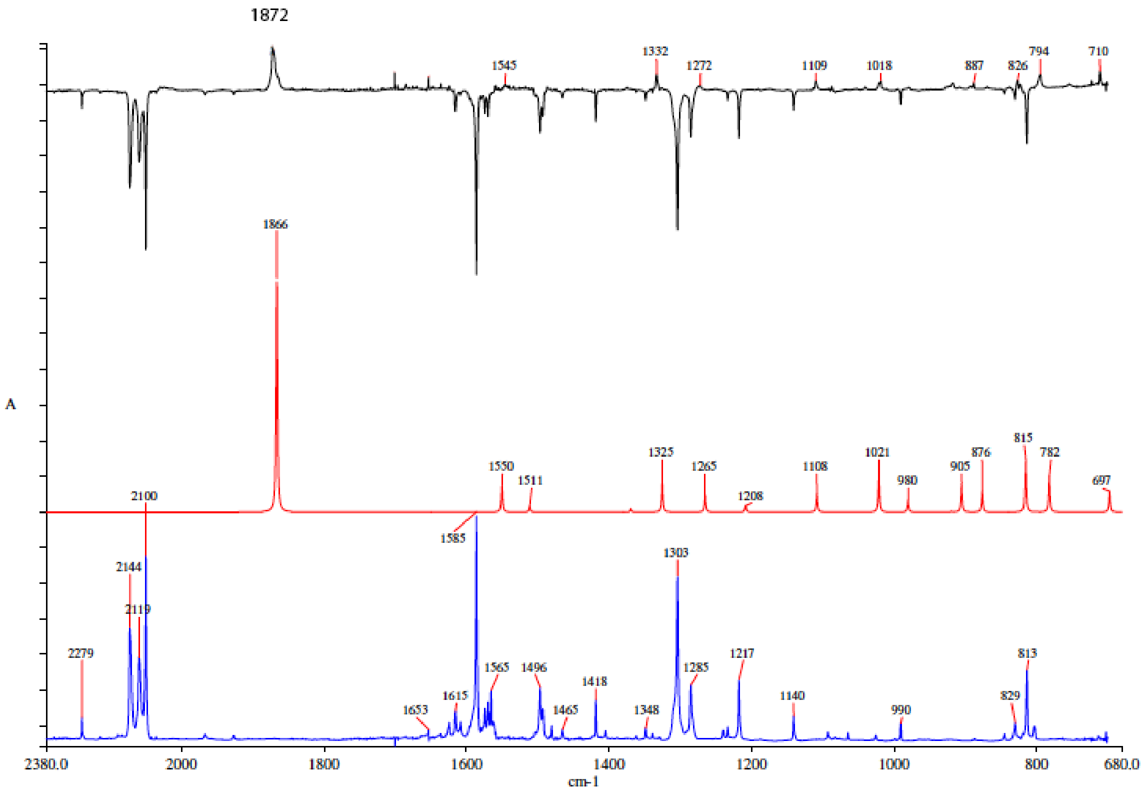

Figure S3: Top: IR-difference spectrum showing **20** (positive peaks) formed on photolysis of **18** (negative peaks) (broadband, 1 min; compare Figure 2). Middle: calculated IR spectrum of **20** (B3LYP/6-3G* with wavenumbers scaled by 0.9613). Bottom: matrix IR spectrum of azide **18** (cf. Figure S2).

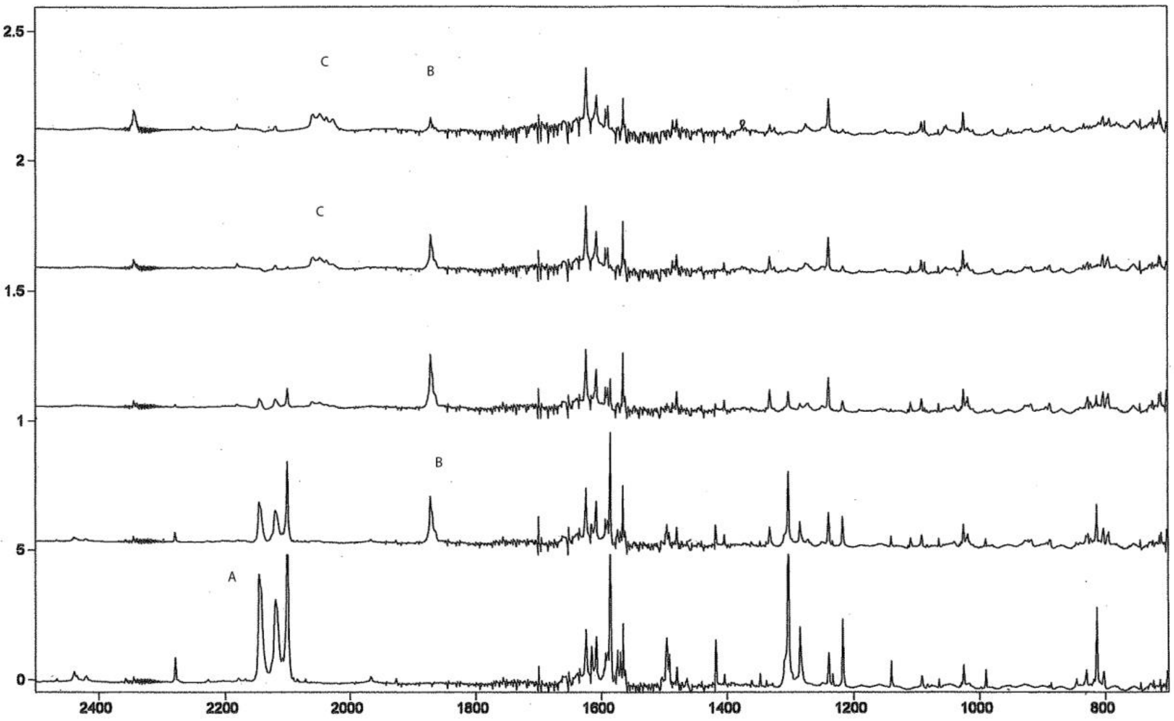

Figure S4: Evolution of the IR spectrum of 4-azidopyridine (**18**, A), matrix isolated in Ar at 7 K, upon photolysis at 290 nm (15 min intervals). The initial spectrum at the bottom is due to 4-azidopyridine (**18**, A). B = **20**. C = **27**. A peak at 2340 cm^{-1} is due to CO_2. Abscissa in cm^{-1}.

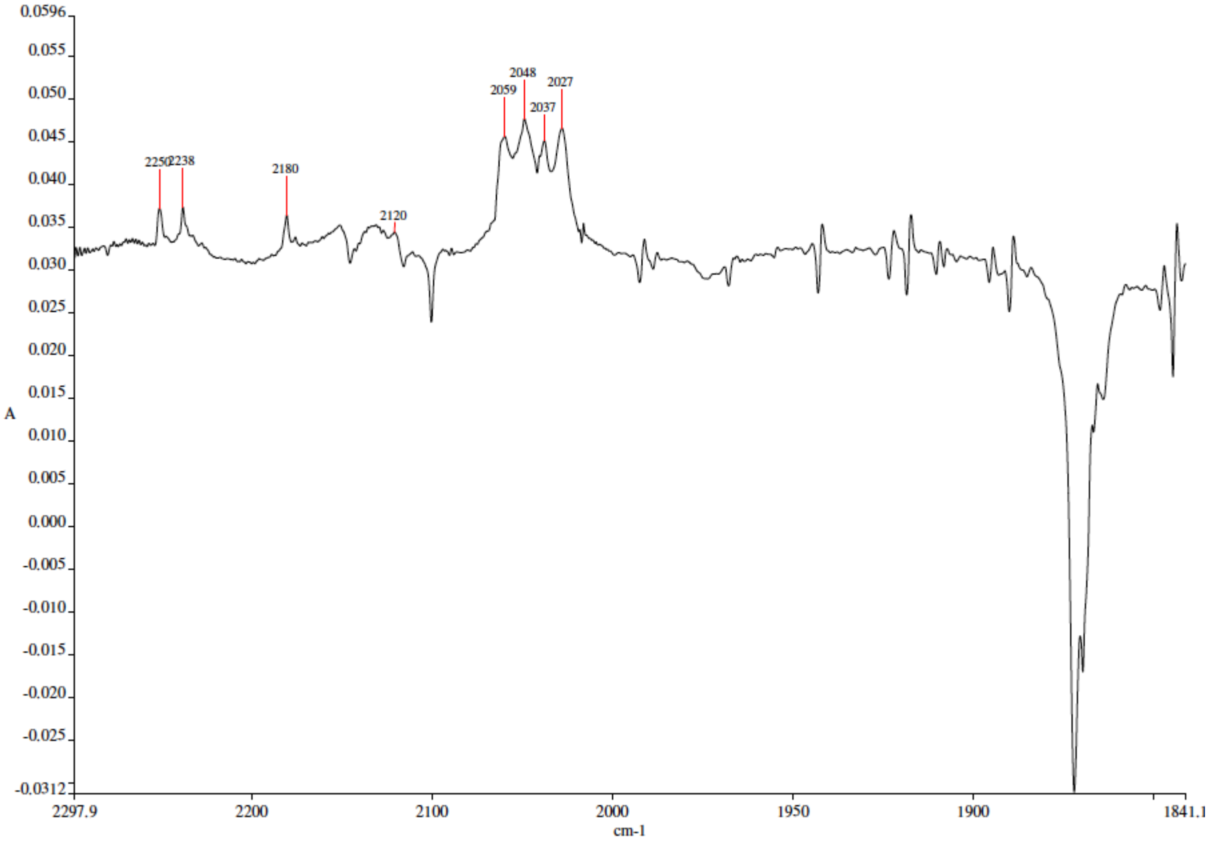

Figure S5: IR-difference spectrum resulting from the 254 nm-photolysis of **20** (negative peak at 1872 cm^{-1}) to **27** (positive peaks at 2027–2059 and 2120 cm^{-1}).

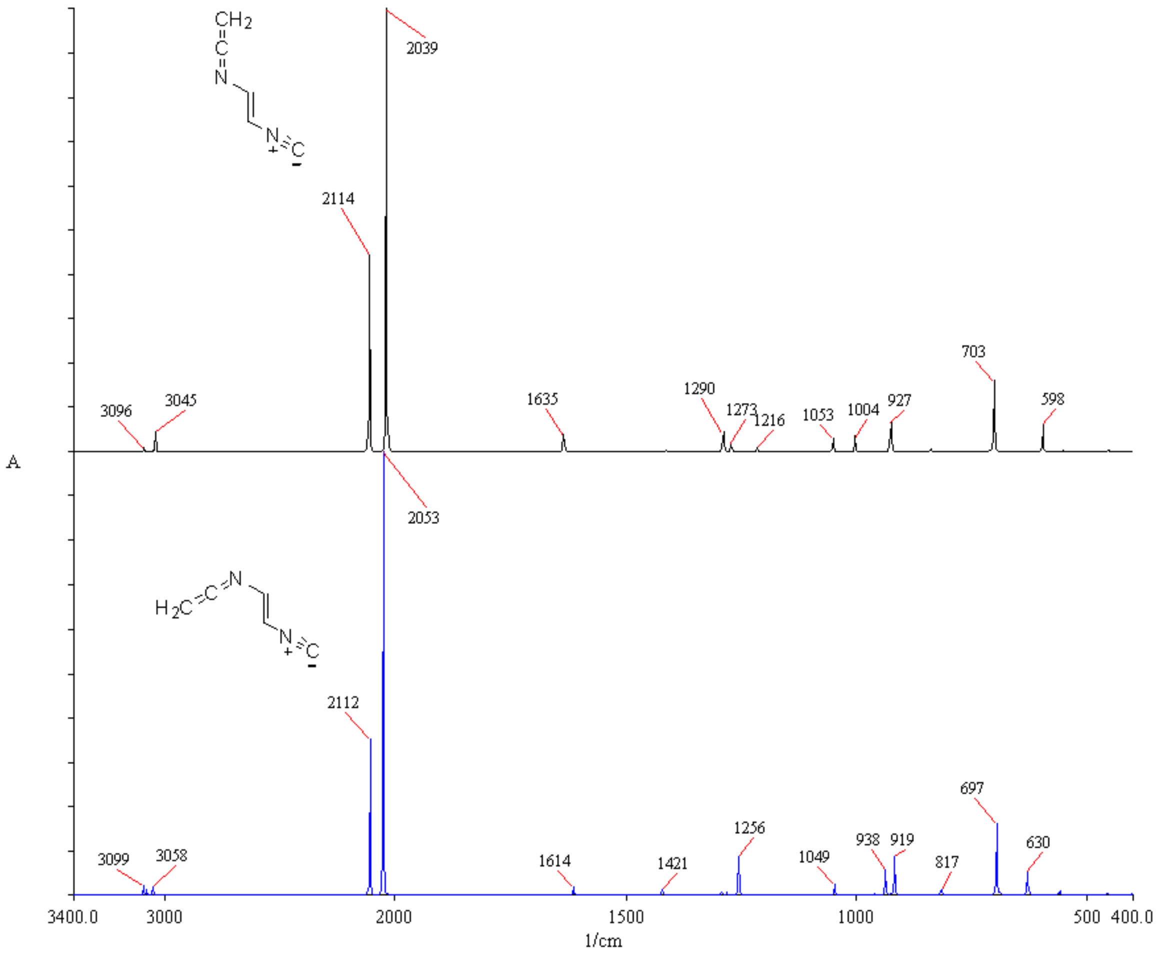

Figure S6: Calculated IR spectra of two *E*-conformers of *N*-(isocyanovinyl)ketenimine (**27**) at the B3LYP/6-31G* level.

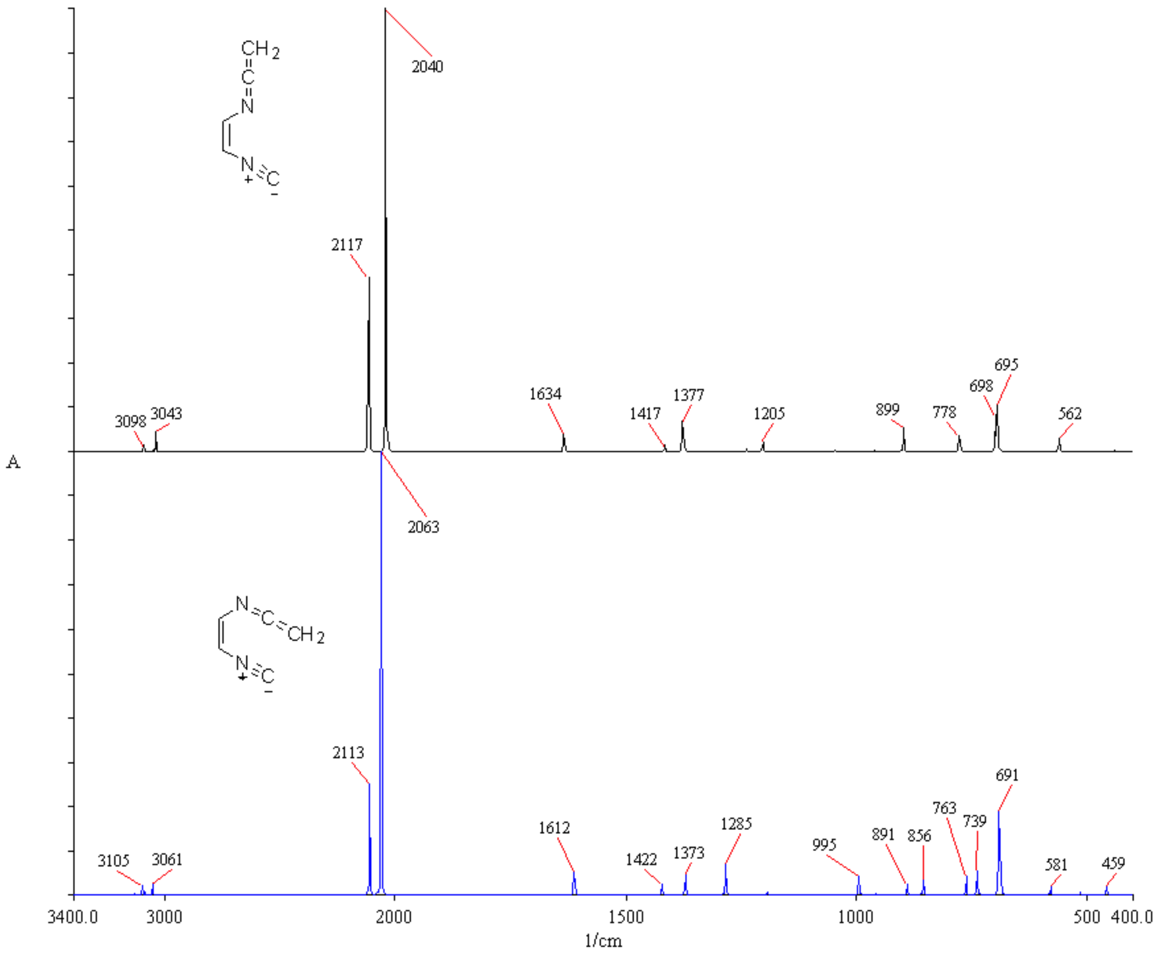

Figure S7: Calculated IR spectra of two *Z*-conformers of *N*-(isocyanovinyl)ketenimine (**27**) at the B3LYP/6-31G* level.

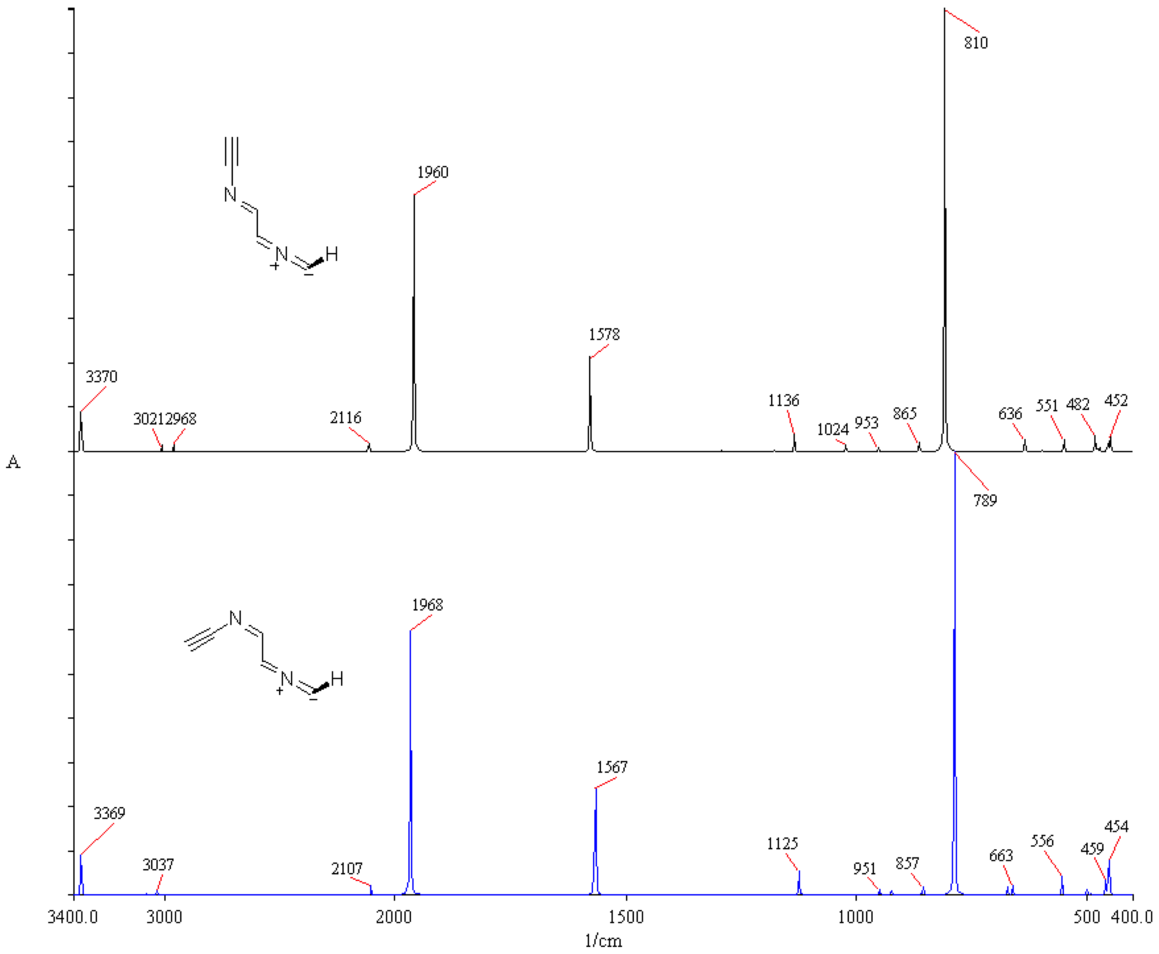

Figure S8: Calculated IR spectra of two s-*E*-conformers of ethynylimino-nitrile ylide **26** at the B3LYP/6-31G* level.

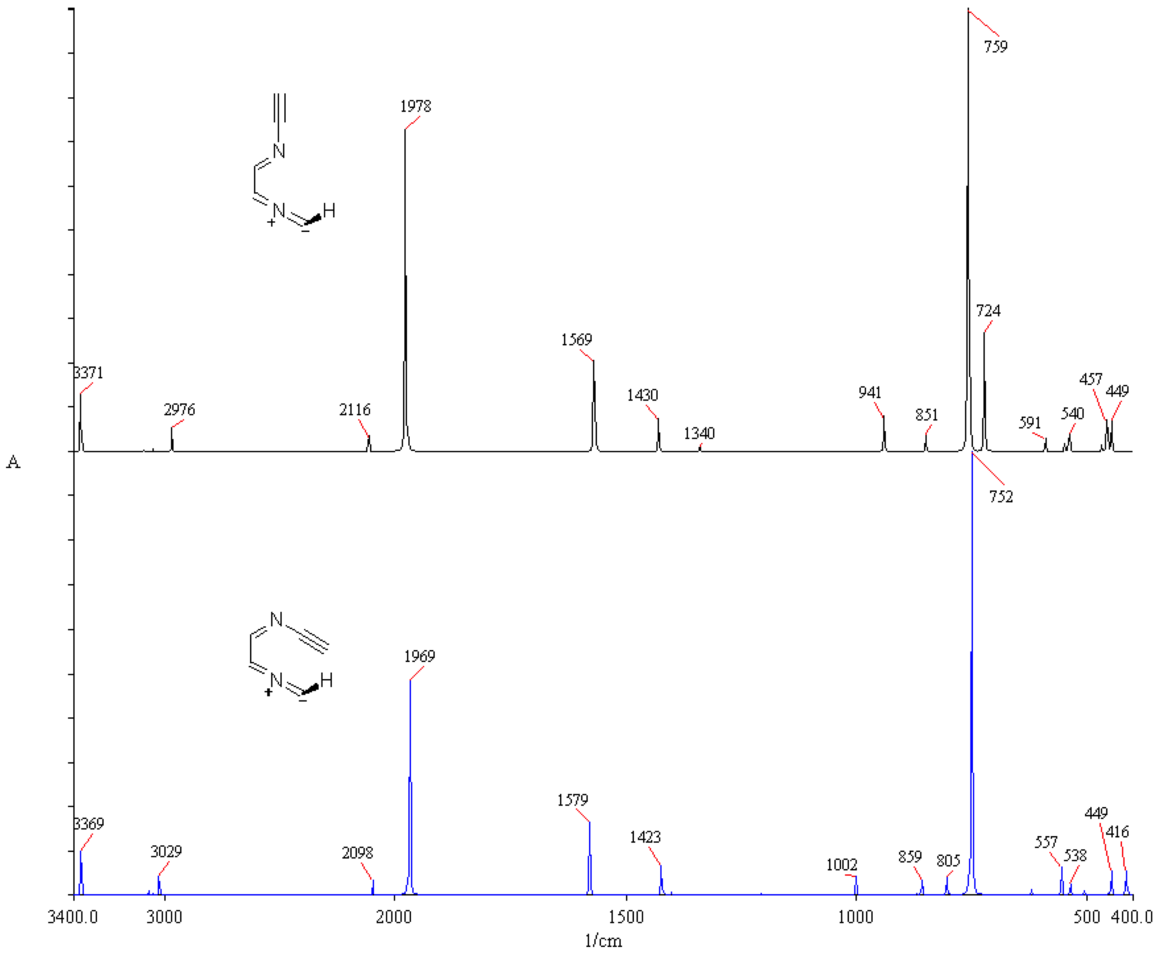

Figure S9: Calculated IR spectra of two s-*Z*-conformers of ethynylimino-nitrile ylide **26** at the B3LYP/6-31G* level.

Computational method

All calculations were carried out at the B3LYP/6-31G* level using the Gaussian 09 program package [1]. Calculated wavenumbers were scaled by a factor of 0.9613 [2].

Ketenimine 20

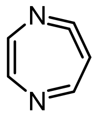

B3LYP/6-31G*; Gaussian 09, Revision A.02

Point group: C1

State= 1-A

HF= -302.3202267 Hartree

Zero-point correction= 0.079815 (Hartree/Particle)

Sum of electronic and zero-point Energies= -302.240411 Hartree

Atom type	Coordinates (angstroms):		
	X	Y	Z
N	1.076995	-1.115559	-0.458882
C	1.558144	0.086766	0.135938
C	0.744691	1.175217	0.245762
N	-0.554515	1.345285	-0.220658
C	-1.474141	0.440080	-0.201807
C	-1.274544	-0.914127	0.373489
C	-0.064403	-1.307750	0.014663
H	2.614010	0.132604	0.388109
H	1.196468	2.101156	0.596477
H	-2.432137	0.702039	-0.657669
H	-1.974185	-1.424993	1.021599

Vibrational frequencies (scaled by 0.9613):

ModeNr.	Symmetry	Wavenumber	Abs.Int.	Rel.Int.
1	A	284.6	11.5	7
2	A	315.6	6.8	4
3	A	382.0	10.7	6
4	A	464.7	2.5	2
5	A	508.4	7.4	4
6	A	595.9	5.6	3
7	A	639.9	53.3	32
8	A	697.7	14.6	9
9	A	783.0	25.7	16
10	A	815.6	35.6	22
11	A	876.5	14.8	9
12	A	906.0	20.0	12
13	A	919.9	0.4	0
14	A	980.7	8.3	5
15	A	1021.0	22.7	14
16	A	1108.7	13.1	8
17	A	1208.8	4.4	3
18	A	1265.8	12.9	8
19	A	1325.3	21.4	13
20	A	1369.4	2.3	1
21	A	1511.1	4.6	3
22	A	1550.1	18.4	11
23	A	1866.9	164.9	100
24	A	3012.0	36.5	22
25	A	3055.5	5.4	3
26	A	3086.1	24.1	15
27	A	3127.9	1.0	1

***(s-Z,Z)*-27**

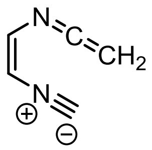

B3LYP/6-31G*; Gaussian 09, Revision A.02

Point group: C1

State= 1-A

HF= -302.3120642 Hartree

Zero-point correction= 0.076249 (Hartree/Particle)

Sum of electronic and zero-point Energies= -302.235815 Hartree

Atom type	Coordinates (angstroms): X	Y	Z
N	-0.797732	-1.057148	-0.426327
C	0.448754	-1.431050	0.069062
C	1.517042	-0.627149	0.226883
N	1.487921	0.728871	0.008602
C	1.495099	1.897647	-0.172373
C	-2.360276	0.837821	0.206055
C	-1.513485	-0.120892	-0.077527
H	0.571960	-2.498466	0.221113
H	2.479106	-1.038143	0.513661
H	-2.220251	1.828687	-0.213159
H	-3.184939	0.667589	0.889865

Vibrational frequencies (scaled by 0.9613):

ModeNr.	Symmetry	Wavenumber	Abs.Int.	Rel.Int.
1	A	63.6	8.2	2
2	A	112.4	9.0	2
3	A	151.0	3.1	1
4	A	272.4	3.5	1
5	A	309.3	5.6	1
6	A	346.9	4.7	1
7	A	459.9	8.4	2
8	A	516.5	3.6	1
9	A	581.8	7.3	2
10	A	691.5	89.0	19
11	A	738.6	25.5	5
12	A	763.8	19.8	4
13	A	856.4	16.0	3
14	A	891.3	10.8	2
15	A	960.7	1.0	0
16	A	995.4	18.9	4
17	A	1193.6	3.8	1
18	A	1285.0	32.1	7
19	A	1372.9	21.0	4
20	A	1422.3	11.7	2
21	A	1612.0	24.3	5
22	A	2063.8	469.3	100
23	A	2113.1	117.6	25
24	A	3061.8	10.9	2
25	A	3089.9	3.5	1
26	A	3106.5	10.3	2
27	A	3141.9	1.1	0

***(s-E,Z)*-27**

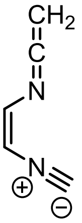

B3LYP/6-31G*; Gaussian 09, Revision A.02

Point group: C1

State= 1-A

HF= -302.3141699 Hartree

Zero-point correction= 0.076284 (Hartree/Particle)

Sum of electronic and zero-point Energies= -302.237886 Hartree

Atom type	Coordinates (angstroms): X	Y	Z
N	-0.675587	-0.236777	-0.000602
C	0.085046	0.934751	-0.000282
C	1.429520	0.909343	0.000474
N	2.175094	-0.240478	0.000260
C	2.855079	-1.208262	0.000119
C	-3.215597	-0.338753	0.001138
C	-1.909558	-0.235316	-0.002036
H	-0.423760	1.897876	-0.000429
H	1.996906	1.834545	0.000778
H	-3.771047	-0.381235	-0.930485
H	-3.765586	-0.380976	0.936051

Vibrational frequencies (scaled by 0.9613):

ModeNr.	Symmetry	Wavenumber	Abs.Int.	Rel.Int.
1	A	73.3	4.5	1
2	A	93.9	3.8	1
3	A	179.2	2.4	0
4	A	242.3	0.1	0
5	A	303.0	0.8	0
6	A	351.9	0.2	0
7	A	442.2	1.3	0
8	A	563.0	17.6	3
9	A	586.8	0.7	0
10	A	695.6	55.5	10
11	A	699.8	43.8	8
12	A	779.0	20.4	4
13	A	899.7	31.7	6
14	A	910.3	0.1	0
15	A	961.4	1.7	0
16	A	1048.6	2.2	0
17	A	1205.4	11.6	2
18	A	1239.4	4.5	1
19	A	1377.5	38.3	7
20	A	1417.8	7.0	1
21	A	1634.2	22.7	4
22	A	2039.9	563.0	100
23	A	2117.3	221.2	39
24	A	3043.4	25.5	5
25	A	3054.8	4.7	1
26	A	3097.9	7.6	1
27	A	3131.7	0.3	0

***(s-Z,E)*-27**

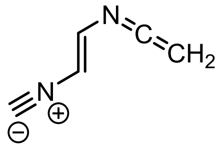

B3LYP/6-31G*; Gaussian 09, Revision A.02

Point group: C1

State= 1-A

HF= -302.3116449 Hartree

Zero-point correction= 0.076100 (Hartree/Particle)

Sum of electronic and zero-point Energies= -302.235545 Hartree

Atom type	Coordinates (angstroms): X	Y	Z
N	1.155734	0.946332	-0.188781
C	-0.213239	0.835197	0.075069
C	-0.944008	-0.263119	-0.185442
N	-2.303462	-0.299802	0.004641
C	-3.473615	-0.377178	0.160907
C	3.009043	-0.757279	0.138540
C	2.015369	0.083707	-0.017759
H	-0.682313	1.747502	0.429549
H	-0.497084	-1.171118	-0.580239
H	3.241338	-1.487793	-0.629598
H	3.610856	-0.742276	1.041380

Vibrational frequencies (scaled by 0.9613):

ModeNr.	Symmetry	Wavenumber	Abs.Int.	Rel.Int.
1	A	74.2	2.1	0
2	A	104.5	2.5	0
3	A	165.0	8.8	2
4	A	215.7	9.0	2
5	A	336.3	8.1	2
6	A	365.5	4.7	1
7	A	403.4	3.5	1
8	A	458.1	2.6	0
9	A	561.1	5.3	1
10	A	630.5	27.5	5
11	A	697.6	84.1	16
12	A	817.2	6.9	1
13	A	919.4	45.2	9
14	A	938.9	29.8	6
15	A	964.1	1.8	0
16	A	1049.8	13.2	3
17	A	1256.3	45.7	9
18	A	1281.1	4.5	1
19	A	1292.7	3.3	1
20	A	1421.4	7.8	1
21	A	1614.1	9.3	2
22	A	2053.3	526.5	100
23	A	2113.0	184.6	35
24	A	3058.2	8.3	2
25	A	3084.0	6.8	1
26	A	3099.7	11.0	2
27	A	3136.2	0.5	0

(s-E,E)-27

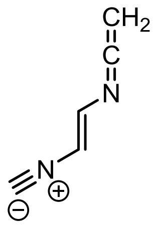

B3LYP/6-31G*; Gaussian 09, Revision A.02

Point group: C1

State= 1-A

HF= -302.3159179 Hartree

Zero-point correction= 0.076070 (Hartree/Particle)

Sum of electronic and zero-point Energies= -302.239848 Hartree

Atom type	Coordinates (angstroms): X	Y	Z
N	1.073933	0.609255	-0.000226
C	-0.124455	-0.111947	-0.001177
C	-1.289921	0.556533	0.000538
N	-2.503890	-0.082166	0.000362
C	-3.574639	-0.585496	0.000211
C	3.403243	-0.409702	0.001269
C	2.183472	0.067686	-0.001867
H	-0.096723	-1.201192	-0.002838
H	-1.316071	1.641931	0.002260
H	3.921119	-0.616209	-0.930193
H	3.915173	-0.616591	0.935971

Vibrational frequencies (scaled by 0.9613):

ModeNr.	Symmetry	Wavenumber	Abs.Int.	Rel.Int.
1	A	93.6	0.0	0
2	A	98.1	0.9	0
3	A	182.2	9.0	2
4	A	198.3	6.0	1
5	A	357.9	0.9	0
6	A	364.5	3.4	1
7	A	394.3	1.0	0
8	A	454.9	1.8	0
9	A	555.7	1.1	0
10	A	598.9	31.5	6
11	A	703.8	86.3	16
12	A	840.1	3.6	1
13	A	927.2	34.7	6
14	A	961.4	0.5	0
15	A	1004.2	18.1	3
16	A	1053.6	16.9	3
17	A	1216.5	6.1	1
18	A	1273.3	8.0	2
19	A	1290.7	23.5	4
20	A	1415.5	2.7	0
21	A	1635.3	19.6	4
22	A	2038.7	535.5	100
23	A	2114.6	238.5	45
24	A	3045.4	24.3	5
25	A	3053.8	4.2	1
26	A	3095.8	5.4	1
27	A	3130.4	0.3	0

References

1. Gaussian 09, Revision A.02, Frisch, M. J.; Trucks, G. W.; Schlegel, H. B.; Scuseria, G. E.; Robb, M. A.; Cheeseman, J. R.; Scalmani, G.; Barone, V.; Mennucci, B.; Petersson, G. A.; Nakatsuji, H.; Caricato, M.; Li, X.; Hratchian, H. P.; Izmaylov, A. F.; Bloino, J.; Zheng, G.; Sonnenberg, J. L.; Hada, M.; Ehara, M.; Toyota, K.; Fukuda, R.; Hasegawa, J.; Ishida, M.; Nakajima, T.; Honda, Y.; Kitao, O.; Nakai, H.; Vreven, T.; Montgomery, Jr., J. A.; Peralta, J. E.; Ogliaro, F.; Bearpark, M.; Heyd, J. J.; Brothers, E.; Kudin, K. N.; Staroverov, V. N.; Kobayashi, R.; Normand, J.; Raghavachari, K.; Rendell, A.; Burant, J. C.; Iyengar, S. S.; Tomasi, J.; Cossi, M.; Rega, N.; Millam, J. M.; Klene, M.; Knox, J. E.; Cross, J. B.; Bakken, V.; Adamo, C.; Jaramillo, J.; Gomperts, R.; Stratmann, R. E.; Yazyev, O.; Austin, A. J.; Cammi, R.; Pomelli, C.; Ochterski, J. W.; Martin, R. L.; Morokuma, K.; Zakrzewski, V. G.; Voth, G. A.; Salvador, P.; Dannenberg, J. J.; Dapprich, S.; Daniels, A. D.; Farkas, Ö.; Foresman, J. B.; Ortiz, J. V.; Cioslowski, J.; Fox, D. J. Gaussian, Inc., Wallingford CT, 2009.
2. Wong, M. W. *Chem. Phys. Lett.* **1996**, *256*, 16502.
